# The frequency and types of resident relocations in Dutch nursing homes: a nationwide cohort study of electronic health record data

**DOI:** 10.1007/s41999-024-01064-0

**Published:** 2024-10-01

**Authors:** Miranda C. Schreuder, Karlijn J. Joling, Wim G. Groen, Marieke Perry, Elleke G. M. Landeweer, Hendrika J. Luijendijk, Sytse U. Zuidema, the RELOCARE Consortium, H. Verbeek, J.P.H. Hamers, J.M.G.A. Schols, B. de Boer, J.H.J. Urlings, M. Brouwers, W.P. Achterberg, M.A.A. Caljouw, E.G.M. Landeweer, H.J. Luijendijk, M.C. Schreuder, S.U. Zuidema, M Perry, R.T.C.M. Koopmans, K.G. Luijkx, A. Stoop, W.G. Groen

**Affiliations:** 1 Department of Primary- and Long-Term Care, University of Groningen, University Medical Center Groningen, PO Box 196, 9700AD Groningen, The Netherlands; 2 Department of Medicine for Older People, Amsterdam UMC, Location Vrije Universiteit Amsterdam, De Boelelaan 1117, Amsterdam, The Netherlands; 3 Amsterdam Public Health, Ageing & Later Life, Amsterdam, The Netherlands; 4 Amsterdam Movement Sciences, Ageing & Vitality, Rehabilitation & Development, Amsterdam, The Netherlands; 5 Department of Primary and Community Care, Donders Institute for Brain Cognition and Behaviour, Radboud University Medical Center, Nijmegen, The Netherlands; 6 Department of Care Ethics, University of Humanistic Studies, Utrecht, The Netherlands

**Keywords:** Nursing home, Long-term care, Registration data, Relocations, Residents

## Abstract

**Purpose:**

One third of Dutch nursing home residents relocated at least once during length of stay. Roughly 75 percent were individual relocations and the other 25 percent concerned group relocations. The average yearly number of individual relocations was about 3 times as high in the first 4 months after admission compared with later periods.

**Methods:**

We performed an historical cohort study of pseudonymized registration data from a Dutch electronic health record. We selected data from nursing home residents aged 65 years and older who stayed on a ward for physical impairment or dementia and passed away between 2015 and 2019. Our study sample consisted of 26,060 long-stay nursing home residents from 67 nursing homes in the Netherlands. We examined the number of relocations, trends over time, individual versus group relocations and relocation destinations.

**Results:**

We found that approximately one-third of long-stay nursing home residents relocated at least once with an average of 36 relocations per 100 residents per year. Roughly, 75 percent of relocations were individual relocations and 25 percent concerned group relocations. In the first 4 months after admission, the average number of individual relocations per 100 resident per year was about 3 times as often compared to later periods after admission. Most individual relocations were within the same type of care.

**Conclusion:**

A considerable proportion of Dutch long-stay nursing home residents experienced one or more relocations. Relocations for individual reasons occurred mostly in the first months after admission. Further investigation is warranted to explore which factors lead to relocations.

## Key summary points

**Aim:**

We examined the occurrence of relocations of nursing home residents, the distribution of individual and group relocation types, the distribution of destinations, and trends over time.

**Findings:**

One third of Dutch nursing home residents relocated at least once during length of stay. Roughly 75 percent were individual relocations and the other 25 percent concerned group relocations. The average yearly number of individual relocations was about 3 times as high in the first 4 months after admission compared with later periods.

**Message:**

A considerable proportion of Dutch long-stay nursing home residents experienced one or more relocations.

## Introduction

In 2019, 9.2% of the global population was 65 years or older, while 16.5% of the US and 20.1% of the European Union's population fell into this age group [1]. The percentage of adults aged 65 and over receiving long-term care in institutions varied, ranging from approximately 1–3% in countries like Poland, Portugal, and Japan, to around 12% in countries such as the Netherlands and the Czech Republic, and up to 23–24% in countries like Israel and Switzerland [2].

In the Netherlands, nursing home care is covered by the Long-Term Care Act, which provides for a social insurance scheme. Enrolment is automatic and mandatory, and access to care is a legal right. Eligibility is based on formal criteria regarding an individual’s health status and assessed by an independent assessor from a central agency (CIZ) [3].

When a person is admitted to a nursing home this is typically for the remainder of that person’s life and residing in a permanent location is preferable. Relocations of long-stay nursing home residents still happen for various reasons, for example because of closure, outdated real estate, a change in care needs or preferences of the residents or relatives [4, 5]. In case of closure or outdated real estate, residents may be relocated to a new (temporary) location as a group or can be divided over different existing locations. These relocations are unavoidable and cannot be contested [6, 7]. In the event of a change in care needs or preferences of the residents or relatives, the individual resident may relocate to another ward, location or care organization [6]. In the Netherlands, there are no governmental guidelines for relocations within nursing homes.

The frequency of nursing home resident relocations has not been studied often. Previous studies have focused on the frequency of nursing home closures and opening of new nursing homes. A review found that 5% of nursing homes relocated, merged or closed between 1992 and 1997 in the USA [6]. A subsequent US study reported a 16% closure rate for certified nursing home facilities between 1999 and 2008 [5]. In England, 5% of nursing homes closed and 1% was newly opened in 2000–2001 [8]. Other studies reported a 40% loss of beds between 2004 and 2009 in England [9]. In addition, two others US studies showed that on average 6.5% of residents relocated within a facility per year in 1992–1997 and 7.7% between facilities in 2019 [6, 10]. Unfortunately, these figures are either old, based on a small sample, or stemming from the US only. Detailed insight in the occurrence of nursing home residents’ relocations and studies from Europe are still missing.

Relocations within nursing homes may have negative effects on various health domains (e.g., functional, cognitive and psychological) and mortality of residents [6, 7, 9, 11–14]. Relocating within nursing homes can also be stressful for residents, and they may be unhappy about leaving a familiar place or may feel it is beyond their control [4, 6, 7, 15, 16]. Nevertheless, some residents may be glad to relocate because the new home is closer to relatives or is more luxurious than the old location. Besides, the impact of a relocation on the resident can vary throughout the process [7, 15]. Insights in the occurrence of relocations, distribution of individual and group destinations (type of care; and other wards, locations or organizations) may help to develop policies that could anticipate and prevent negative experiences related to relocations between nursing homes. Therefore, the aim of this study was to examine the occurrence of relocations of nursing home residents, the distribution of individual and group relocations, the distribution of destinations, and trends over time.

### Methods

We performed a historical cohort study of pseudonymized routinely collected health care data from Dutch nursing home residents that used the electronic health record (EHR) Ysis [17]. This EHR is used by approximately half of the organizations for long-term care in the Netherlands.

The Medical Ethics Review Committee of VU University Medical Center approved this study and considered it not to be subject to the Dutch Medical Research Involving Human Subjects Act. Because pseudonymized data of deceased persons collected for routine care purposes were used, informed consent of patients was not obtained. Patients were informed by their healthcare provider about the use of their pseudonymized EHR data and could object.

### Study sample

On the extraction date January 19th, 2022, the Ysis database contained the data of 495,364 patients of which 102,824 patients opted-out for scientific research or received care from an organization that opted-out for scientific research. We selected long-stay nursing home residents aged 65 years and older who resided on a ward for physical impairment or dementia and passed away between 2015 and 2019. We chose to include only deceased residents, as this facilitated the data request process given privacy regulations in the Netherlands. To avoid COVID-19-related relocations, we excluded residents who were alive from 2020 onwards. Short-term stays, e.g. geriatric rehabilitation prior to long-term stays, were excluded, but intermitted short-term stay was not an exclusion criterium. Patients admitted to a nursing home before the start of Ysis were excluded because the complete length of stay could not be calculated. As the application of these criteria was in part performed by the data manager of Ysis, we could not provide a detailed breakdown of selected participants by each criterion.

### Data retrieval

We obtained the following demographic resident characteristics: sex, birth year and date of death. Due to privacy concerns, it was not possible to obtain geographical information of the residents. Nevertheless, we know that Ysis is used throughout the Netherlands, with a concentration in the western part of the country. To identify the nature and type of relocation, we obtained the following residents relocation variables: start- and end-date per stay, coded organization ID, coded location ID and ward number. We considered a relocation to a different ward in the same location or to another location within the same or another organization as a relocation within a nursing home. We did not consider transfers within the same ward to another room as a relocation, because the surroundings, fellow residents and care personnel remain the same, and can be expected to have limited negative impact. Also, such room transfers were difficult to distinguish from administrative errors. We considered a relocation as group relocation if at least three residents in the same location had the same end-date.

We defined four types of care: long-term care for dementia, long-term care for physical impairment, long-term care not specified, and (intermittent) short-term care. To be eligible to live in a nursing home in the Netherlands, independent governmental assessors with a background in health care determine the type and volume of care to which an individual is entitled [3]. This entitlement is then formalized into a care profile ranging from 1 to 10. Care profiles 5–8 give access to long-term nursing home care [3, 18]. As the field for type of care often was not filled in or updated, we determined the type of care per stay based on proxy variables, which included standardized billing codes used to identify short-term care; restrictive measures to identify long-term care in general, such as bed bars or wedges; measures to identify dementia care, such as acoustic monitoring or GPS technology; care profiles; historical type of care; and total length of stay.

### Analysis

Descriptive statistics were used to present characteristics of the study sample, and the relocated and non-relocated subgroup. We calculated the percentage of females, mean age at admission (with SD), and median length of stay (with IQR) independently of any relocations. Length of stay was categorized based on the interquartile range as 0–4 months, 4 months-1 year, 1–2 years, and 2 years or more. Additionally, we analyzed the percentages of type of care at admission and medical history at death.

The total number of relocations and the number of relocations per 100 residents per year were calculated. In case of two or more relocations per resident, we also calculated the average period in days between the relocations.

Next, we investigated the distribution of group and individual relocations. For the individual relocations, we also plotted the type of care the residents received in the old and new stay in percentages. We also determined which percentage of individual relocations took place to a different ward in the same location, to another location and to another care organization.

As the research project progressed, we decided post-hoc to analyze the data from the first admission of the included residents, which could have occurred between 2010, the introduction year of Ysis, and 2019, and also to examine the trend in relocations over time for individual and group relocations combined and apart. All analyses were performed using IBM SPSS version 28 (IBM Corp, Armonk, NY).

## Results

**Fig. 1:**
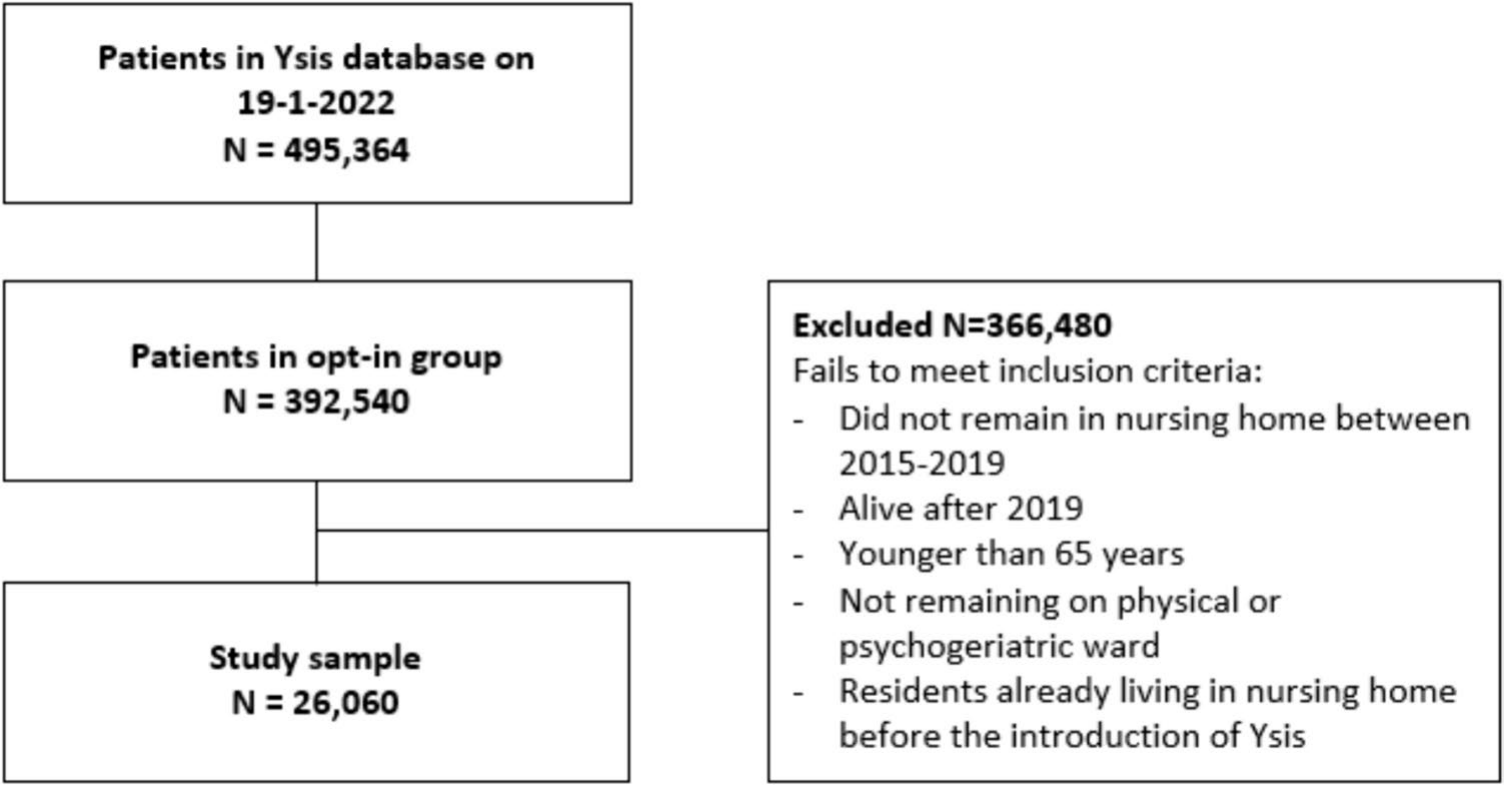
Selection of study sample

Data from 67 different care organizations with 739 different locations (range 1–45 locations per care organization) were available. After application of the inclusion and exclusion criteria, we had a study sample of 26,060 long-stay nursing home residents (Fig. 1). A small proportion concerned intermittent short stays (2.6%). For some long-term stays, it was not evident whether they involved care for dementia or physical impairment (13.5%). For a small proportion of intermittent stays (0.4%), the type of care could not be determined at all.

### Sample characteristics

The mean age of the study sample at admission was 84.6 years (see Table 1). Almost 62% of the study sample was female. At baseline, 55.9% of residents resided at a ward for dementia, 29.4% at a ward for physical impairment, and for 14.7% it concerned non-specified long-term stay. The median length of stay was 0.97 years (IQR: 0.31;2.01) independent of relocations. It was 1.65 year (IQR: 0.77;2.80) for relocated residents, and 0.71 year (IQR: 0.21;1.60) for non-relocated residents. At death, 69.3% of relocated residents and 59.9% of residents who did not relocate had a diagnosis of dementia registered.

**Table 1:** Sample characteristics

	Relocated group (*n* = 8,312)	Non-relocated group (*n* = 17,748)	All residents (*n* = 26,060)
Sex, % females	61.1	62.1	61.8
Age at admission, years ± SD	83.7 ± 7.2	85.0 ± 7.2	84.6 ± 7.2
Median length of nursing home stay, years (IQR)	1.65 (0.77;2.80)	0.71 (0.21;1.60)	0.97 (0.31;2.01)
Length of stay, %			
Up to 4 months	11.2	32.7	25.8
4 months to 1 year	20.8	26.9	25.0
1–2 year	27.0	22.5	23.9
2 year or more	41.0	17.9	25.3
Type of long-term care at admission, %			
For dementia	49.3	59.0	55.9
For physical impairment	28.8	29.7	29.4
Unspecified	21.9	11.4	14.7
Medical history at death, %			
Dementia	69.3	59.9	62.9
Parkinson’s disease, Korsakov syndrome or Huntington’s disease	8.8	7.6	8.0
Cerebrovascular disease	37.2	34.5	35.4
Cardiovascular disease	47.6	46.0	46.5
Pulmonary disease	19.2	18.1	18.4
Kidney failure	20.5	19.8	20.0
Diabetes mellitus	19.3	18.0	18.4

### Relocations

In total, 11,945 relocations took place, which corresponded with 36 relocations per 100 residents per year. Of all residents, 22.4% relocated once during their nursing home stay, 6.6% twice and 2.9% three times or more. For the residents with two or more relocations (9.5% of residents), there was an average of 223 days between the relocations (SD: 292).

Of all relocations, 74.8% were individual relocations and 25.2% were group relocations. Most individual relocations occurred within long-term care for dementia (43.3%) and physical impairment (16.5%), as shown in Table 2. Of the individual relocations, 48.5% took place within the same location, 44.1% to another location and 7.4% to another care organization.

**Table 2:** Type of care of individual relocations (in percentages)

From	To					
	Long-term dementia	Long-term physical impairment	Long-term care, unspecified*	Intermitted short-stay	Unknown^†^	Total
Long-term dementia	43.3	2.2	2.1	1.6	0.5	49.8
Long-term physical impairment	7.8	16.5	2.0	3.0	0.3	29.6
Long-term care, unspecified*	7.8	3.5	5.2	2.5	0.5	19.5
Intermitted short-stay	0.0	0.0	0.0	0.0	0.0	0.0
Unknown†	0.5	0.3	0.2	0.0	0.2	1.2
Total	59.3	22.6	9.5	7.2	1.5	100.0

*Long-term stay, but unknown whether the care was for dementia or physical impairment

^†^For a small proportion of intermittent stays the type of care could not be determined

Table 3 shows the number of relocations per 100 residents per year plotted against the period after admission and calendar year for all relocations combined (3a), individual relocations (3b) and group relocations (3c)*.* The number of group relocations per 100 residents per year was stable for each period after admission. In contrast, the average number of individual relocations was on average three times as high in the 4 months after admission compared with later. The average yearly number of individual and group relocations combined varied slightly over the calendar years from 31 to 43 per 100 residents, but without a clear trend.

**Table 3:** Number of relocations per 100 residents per year

Period after admission	2010 (*n* = 98)	2011 (*n* = 391)	2012 (*n* = 1,525)	2013 (*n* = 4,389)	2014(*n* = 8,184)	2015 (*n* = 15,747)	2016 (*n* = 20,327)	2017 (*n* = 22,809)	2018 (*n* = 21,676)	2019 (*n* = 13,837)	Average*
A) Individual and group relocations combined											
0 to 4 months	0	63	57	64	59	62	68	60	64	88	65
4 to 12 months		14	44	33	34	23	30	29	34	36	30
1 to 2 years		0	27	31	31	27	21	21	29	33	26
≥ 2 years			17	32	51	23	28	22	28	26	27
Average†	0	31	43	43	41	34	35	31	36	41	36
B) Individual relocations											
0 to 4 months	0	53	55	51	52	55	56	56	59	85	58
4 to 12 months		14	34	24	21	18	20	20	24	32	22
1 to 2 years		0	21	21	20	16	13	13	17	28	16
≥ 2 years			17	26	20	16	16	14	15	21	16
Average†	0	27	37	33	28	26	25	24	26	36	27
C) Group relocations											
0 to 4 months	0	9	2	13	7	7	12	5	5	3	7
4 to 12 months		0	10	9	13	5	10	9	10	4	9
1 to 2 years		0	6	10	12	11	8	8	12	5	9
≥ 2 years			0	6	30	8	13	8	14	5	11
Average†	0	3	6	10	13	8	10	8	11	4	9

*weighted for all calendar years; †weighted for all periods after admission

## Discussion

Our study, based on routine care data from Dutch nursing homes, showed that approximately one third of long-stay nursing home residents relocated at least once to another ward, location, or organization during their nursing home stay, with an average of 36 relocations per 100 residents per year. Roughly, 75 percent of relocations were individual relocations, the other 25 percent concerned a group relocation. The average yearly number of individual relocations per 100 residents is about 3 times as high in the first 4 months after admission compared with later periods. Most individual relocations were not related to changes in type of care.

### Frequency of relocations

We found an average of 36 relocations per 100 nursing home residents per year, of which on average 9 were group relocations. We had to distinguish between group and individual relocations based on the simultaneous occurrence of relocations of other residents. Hence, if a group of residents relocated one by one, this group relocation will incorrectly have been labeled as individual relocations, resulting in an underestimation of the number of group relocations.

Previous studies about group relocations reported closure rates of nursing homes of 5–16% and loss of beds of 40% [5, 8, 9]. Comparison with our findings is difficult for two reasons. First, closure is just one reason for group relocations, next to renovation of outdated real estate. Second, the number of nursing homes that close does not provide insight into the number of residents who therefore need to relocate. Although previous studies found that the rate of nursing home closures was increasing [7, 9], we did not find an increase in group relocations over the years.

We found an average of 27 individual relocations per 100 residents per year. This is higher than the 7.7 individual first relocations to another home per 100 residents during 10 months in an American study [10]. Our figure also includes relocations within the same location (48.5% of individual relocations) and the residents who experience two or more relocations (9.5%).

It is a notable and new finding that individual relocations within nursing homes occur approximately three times more often in the first months than in later periods. While there is no existing literature about this phenomenon, we have a few potential explanations for the observed phenomenon. First, it regularly happens that residents are admitted to a nursing home following an acute crisis [20, 21]. In the Netherlands, some people opt not to be on the waiting list for a nursing home, because if they were, the care they receive at home is no longer fully reimbursed [22]. The consequence of an admission due to a crisis is that the resident often is not admitted to the location or ward that best suits the residents’ needs or preference, because there was no free room available. This may result in the need for a subsequent relocation within the nursing home or to another nursing home.

Second, an admission to the nursing home is a major life event for residents, even if it did not occur out of crisis. The admission can lead to prolonged mental and physical health consequences and even to relocation stress syndrome [23, 24]. Deterioration in the period after admission may result in the need for a subsequent relocation to a ward that better suits the newly developed residents’ needs.

Third, before an admission to the nursing home, assessing the type of care that the (future) resident will require in the nursing home may be challenging. This can also lead to the resident not being admitted to the most appropriate ward at once.

We found that around two thirds of individual relocations occurred within the same type of care, with a notable 43.3 percent specifically within long-term care for dementia. Even though we were unable to determine the reasons for these relocations, it is possible that they included relocations from a regular dementia ward to a dedicated special care unit [25].

Even with knowledge of the incidence of various types of relocations within nursing homes, the question remains how desirable these relocations are. Relocations within nursing homes have a potential negative effect on functional, cognitive and psychological health domains [6, 7, 9, 11]. In the USA, nursing home closures increased the distance to nursing homes and hospitals for rural residents, with the hardest hit for areas with a higher concentration of ethnic groups and poverty [5, 26]. However, some studies also reported positive effects, such as an improvement in daily functioning and lower levels of anxiety and depressive symptoms (7, 15). Nonetheless, it is hard to predict which residents will experience the negative and positive consequences.

### Strengths and limitations

Using a large dataset is one of the main strengths of this study. Utilizing EHR data from Ysis, which is used by more than half of the Dutch nursing homes, also offered the advantage of studying an unselected cohort, representative of the Dutch nursing home population.

However, as the EHR has been designed for healthcare providers and not primarily for scientific research, it remains unclear whether missing data represent events that did not occur or that were undocumented. Another downside of using an EHR is the lack of visibility into relocations from facilities with other EHRs to a facility using EHR Ysis. This could have led to an underestimation of the total amount of relocations and the length of stay of residents. Furthermore, we had to determine the type of care per relocation based on proxy variables.

### Conclusions and implications

This study showed that on average one third of Dutch nursing home residents are relocated during their long-term stay. Roughly 75 percent were individual relocations and the other 25 percent concerned a group relocation. The number of individual relocations is especially high in the first 4 months after admission. Further investigation is warranted to explore the reasons for individual relocations, the experiences and health consequences, and the relationship between availability of nursing homes and other determinants with the number of relocations.

## Data Availability

The data set used for this study is available in a safe digital environment of Amsterdam UMC. Requests to access the data set should be directed to Karlijn Joling (k.joling@amsterdamumc.nl).

## References

[1] Elderly population. https://data.oecd.org/pop/elderly-population.htm. Accessed 29 Aug 2024

[2] Access to long-term care. https://www.oecd-ilibrary.org/sites/4c4694a2-en/index.html?itemId=/content/component/4c4694a2-en. Accessed 2021

[3] Bakx P, Schut E, Wouterse B; Price setting in long-term care in the Netherlands. Marketing & Communicatie ESHPM 2020:5–34.

[4] de Boer B, Caljouw M, Landeweer E et al (2021) The need to consider relocations WITHIN long-term care. J Am Med Directors Assoc. 10.1016/j.jamda.2021.11.021. 10.1016/j.jamda.2021.11.021 34932987

[5] Feng Z, Lepore M, Clark MA et al (2011) Geographic concentration and correlates of nursing home closures: 1999–2008. Arch Intern Med 171(9):806–813. 10.1001/archinternmed.2010.492 21220642 10.1001/archinternmed.2010.492 PMC3748956

[6] Castle NG; Relocation of the elderly (2001) Med Care Res Rev 58(3):291–333. 10.1177/107755870105800302 11523292 10.1177/107755870105800302

[7] Weaver RH, Roberto KA, Brossoie N (2020) A scoping review: characteristics and outcomes of residents who experience involuntary relocation. Gerontologist 60(1):e20–e37. 10.1093/geront/gnz035 31112600 10.1093/geront/gnz035

[8] Netten A, Darton R, Williams J; The Rate, Causes and Consequences of Home Closures. 2002.

[9] Jolley D, Jefferys P, Katona C, Lennon S (2011) Enforced relocation of older people when care homes close: a question of life and death? Age Ageing 40(5):534–537. 10.1093/ageing/afr052 21642641 10.1093/ageing/afr052

[10] Chang CH, Park P, Bynum JP, Montoya A (2023) Nursing home to nursing home transfers during the early COVID-19 pandemic. J Am Med Dir Assoc 24(4):441–446. 10.1016/j.jamda.2023.01.028 36878263 10.1016/j.jamda.2023.01.028 PMC9915045

[11] Holder JM, Jolley D (2012) Forced relocation between nursing homes: residents’ health outcomes and potential moderators. Rev Clin Gerontol 22(4):301–319. 10.1017/S0959259812000147

[12] Ryman FVM, Erisman JC, Darvey LM et al (2019) Health effects of the relocation of patients with Dementia: a scoping review to inform medical and policy decision-making. Gerontologist 59(6):e674–e682. 10.1093/geront/gny031 29718293 10.1093/geront/gny031

[13] Castle NG (2005) Changes in health status subsequent to nursing home closure. Ageing Int 30(3):263–277. 10.1007/s12126-005-1015-x

[14] Montoya A, Park P, Bynum J, Chang CH (2024) Transfer trauma among nursing home residents: development of a composite measure. Gerontologist. 10.1093/geront/gnad085 37392460 10.1093/geront/gnad085

[15] Ibrahim K, Baron S, Lathlean J et al (2022) Moving our care home: A qualitative study of the views and experiences of residents, relatives and staff. Int J Older People Nurs 17(6):e12466. 10.1111/opn.12466 35437921 10.1111/opn.12466 PMC9788319

[16] Falk H, Wijk H, Persson LO (2011) Frail older persons’ experiences of interinstitutional relocation. Geriatr Nurs 32(4):245–256. 10.1016/j.gerinurse.2011.03.002 21601952 10.1016/j.gerinurse.2011.03.002

[17] GeriMedica. [About Ysis]. https://www.gerimedica.nl/over-ysis/ (Date Accessed 2023–12–07 Accessed, date last accessed)

[18] Tjeerdsma A, Mobach M (2013) Relocating a Nursing Home. 12the EuroFM Research Symposium,

[19] CBS. [Number of residents of nursing and care homes 2019]. https://www.cbs.nl/nl-nl/maatwerk/2020/13/aantal-bewoners-van-verzorgings-en-verpleeghuizen-2019 (Date Accessed 2024–08–07 Accessed, date last accessed)

[20] Ryan AA, Scullion HF (2000) Nursing home placement: an exploration of the experiences of family carers. J Adv Nurs 32(5):1187–1195. 10.1046/j.1365-2648.2000.01589.x 11115004 10.1046/j.1365-2648.2000.01589.x

[21] Spang L, Holmefur M, Pettersson C, Lidström-Holmqvist K (2023) Experiences of close relatives of older adults in need of a nursing home: it is we who manage their fragile daily life. Health Soc Care Community 2023:9490086. 10.1155/2023/9490086

[22] [Ik sta op een wachtlijst voor opname in een zorginstelling]. https://www.regelhulp.nl/onderwerpen/wlz/wachtlijst. Accessed 29 Aug 2024

[23] Scott S, Raynor A, Dare J et al (2023) Improving the transition of older adults into residential aged care: a scoping review. Clin Gerontol 10(1080/07317115):2274042 10.1080/07317115.2023.2274042 37929882

[24] Manion PS, Rantz MJ (1995) Relocation stress syndrome: a comprehensive plan for long-term care admissions. Geriatr Nurs 16(3):108–112. 10.1016/s0197-4572(05)80039-4 7782001 10.1016/s0197-4572(05)80039-4

[25] Verhees LHF, Banning LCP, Stalman H et al (2023) 2023 Transferring people with dementia to severe challenging behavior specialized units, an in-depth exploration. Aging Ment Health 10(1080/13607863):2280673 10.1080/13607863.2023.2280673 37993411

[26] Sharma H, Bin Abdul Baten R, Ullrich F et al (2024) Nursing home closures and access to post-acute care and long-term care services in rural areas. J Rural Health. 10.1111/jrh.12822 38225679 10.1111/jrh.12822

